# Predicting protein functions by applying predicate logic to biomedical literature

**DOI:** 10.1186/s12859-019-2594-y

**Published:** 2019-02-08

**Authors:** Kamal Taha, Youssef Iraqi, Amira Al Aamri

**Affiliations:** Department of Electrical and Computer Engineering, Khalifa University, Abu Dhabi, United Arab Emirates

## Abstract

**Background:**

A large number of computational methods have been proposed for predicting protein functions. The underlying techniques adopted by most of these methods revolve around predicting the functions of an unannotated protein *p* from already annotated proteins that have similar characteristics as *p*. Recent Information Extraction methods take advantage of the huge growth of biomedical literature to predict protein functions. They extract biological molecule terms that directly describe protein functions from biomedical texts. However, they consider only *explicitly* mentioned terms that co-occur with proteins in texts. We observe that some important biological molecule terms pertaining functional categories may *implicitly* co-occur with proteins in texts. Therefore, the methods that rely solely on *explicitly* mentioned terms in texts may miss vital functional information *implicitly* mentioned in the texts.

**Results:**

To overcome the limitations of methods that rely solely on explicitly mentioned terms in texts to predict protein functions, we propose in this paper an Information Extraction system called PL-PPF. The proposed system employs techniques for predicting the functions of proteins based on their co-occurrences with *explicitly* and *implicitly* mentioned biological molecule terms that pertain functional categories in biomedical literature. That is, PL-PPF employs a combination of statistical-based *explicit* term extraction techniques and logic-based *implicit* term extraction techniques. The statistical component of PL-PPF predicts *some* of the functions of a protein by extracting the explicitly mentioned functional terms that directly describe the functions of the protein from the biomedical texts associated with the protein. The logic-based component of PL-PPF predicts *additional* functions of the protein by inferring the functional terms that co-occur *implicitly* with the protein in the biomedical texts associated with it. First, the system employs its statistical-based component to extract the explicitly mentioned functional terms. Then, it employs its logic-based component to infer additional functions of the protein. Our hypothesis is that important biological molecule terms pertaining functional categories of proteins are likely to co-occur *implicitly* with the proteins in biomedical texts. We evaluated PL-PPF experimentally and compared it with five systems. Results revealed better prediction performance.

**Conclusions:**

The experimental results showed that PL-PPF outperformed the other five systems. This is an indication of the effectiveness and practical viability of PL-PPF’s combination of explicit and implicit techniques. We also evaluated two versions of PL-PPF: one adopting the complete techniques *(*i.e.*, adopting both the implicit and explicit techniques)* and the other adopting only the explicit terms co-occurrence extraction techniques *(*i.e.*, without the inference rules for predicate logic)*. The experimental results showed that the complete version outperformed significantly the other version. This is attributed to the effectiveness of the rules of predicate logic to infer functional terms that co-occur *implicitly* with proteins in biomedical texts. A demo application of PL-PPF can be accessed through the following link: http://ecesrvr.kustar.ac.ae:8080/plppf/

**Electronic supplementary material:**

The online version of this article (10.1186/s12859-019-2594-y) contains supplementary material, which is available to authorized users.

## Background

Determining protein functions has been one of the central objectives for bioinformaticians, especially after the post-genomic era. This is because proteins have key roles in many biological processes. Identifying protein functions using experimental approaches is laborious and time consuming. Therefore, computational methods have been used extensively as alternatives. The underlying techniques adopted by most of these approaches revolve around computing protein functions from already annotated proteins. Most of them reference already annotated proteins using their structures [22], sequences [33], and/or interaction networks. The key limitation of these approaches is that they require highly reliable predictor algorithms. Recent computational methods exploit the huge growth of biomedical literature to predict protein functions from the information of already annotated proteins that appear within the literature. Some of them extract from the literature texts any information that describes proteins [12]. Others extract only information that describes the functions of proteins [2, 5, 7, 10, 28].

We observe that some important biological molecule terms pertaining functional categories may *implicitly* co-occur with proteins in texts. Therefore, the methods that rely solely on *explicitly* mentioned terms in texts may miss vital functional information *implicitly* mentioned in the texts. Towards this, we propose in this paper an Information Extraction system called PL-PPF (Predicate Logic for Predicting Protein Functions) that employs techniques for predicting the functions of proteins based on their co-occurrences in texts with explicitly and implicitly mentioned biological molecule terms pertaining functional categories. PL-PPF infers the implicit terms using the rules of predicate logic. It does so by triggering protein specification rules recursively in the form of predicate logic’s premises [14]. It extracts the explicit terms by employing Natural Language Processing (NLP) techniques that compute the *semantic relationships* among the biological terms in sentences.

Using known protein and biological characteristics, PL-PPF composes rule-based protein specifications. These specifications are known protein characteristics in literature. PL-PPF composes these specifications in a pattern similar to predicate logic’s premises [14]. It triggers them by applying the standard *inference rules* for predicate logic. It does so to deduce functional relationships between proteins. Ultimately, these deduced relationships enable PL-PPF to predict the functions of unannotated proteins. Let *P*_*u*_ be an unannotated protein. Let *L*_*c*_ be a list of known protein characteristics represented in the form of predicate logic’s premises [14]. PL-PPF would first extract biological molecule terms related to *P*_*u*_ based on their co-occurrences in biomedical texts. It extracts the semantically related biological molecule terms to *P*_*u*_ in the sentences of the texts by employing linguistic computational techniques. It would then utilize these extracted terms as identifiers to serve as triggers for the appropriate premises from the list *L*_*c*_ using the standard rules of inferences [8, 16]. The conclusion of this process is a functional category term that co-occurs implicitly with *P*_*u*_ in the texts.

Similar to our approach, a number of studies employed logic-based approaches as complementary to statistical approaches to perform some biological-related tasks. For example, [20] demonstrated that logic models can be used as complementary to statistical analysis models to identify fundamental properties of molecular networks and to perform biological inferences about the dynamics of intracellular molecular networks. As another example, [21] demonstrated that logic-based approaches are useful for improving static conceptual models in molecular biology. The paper demonstrated that adding logic-based approach can improve the Central Dogma information flow.

Logic-based approaches have been successfully applied to solve complex problems in bioinformatics by viewing these problems as binary classification tasks. For example, [3] achieved acceptable results for predicting protein structures using constraint logic programming techniques. [4] presented a methodology that successfully predicted the tertiary structure of a protein using constraint logic programming. [17] used logic based multi-class classification method to accurately solve the problem of protein fold recognition. It accurately assigned protein domains to folds.

PL-PPF infers the functions of an unannotated protein by going through the following sequential steps:

1. Using known biological characteristics, PL-PPF composes rule-based protein specifications. It composes these specifications in a pattern similar to predicate logic’s premises [14]. “Representing protein specification rules in a pattern similar to predicate logic’s premises” section describes this process in detail.
2. PL-PPF employs computational linguistic techniques to extract the biological molecule terms that are *semantically related* to an unannotated protein *p*_*u*_ based on their explicit co-occurrences in texts. If an extracted term denotes a functional category *f*, PL-PPF will assign *p*_*u*_ the function *f*. PL-PPF will also use the extracted term to serve as a *given premise* and apply it as a trigger identifier for the appropriate protein specification rules to identify additional functions of *p*_*u*_. “Extracting biological molecule terms that cooccur explicitly with an unannotated protein in biomedical texts” section describes this process in detail*.*
3. PL-PPF will assign *p*_*u*_ the functional terms that co-occur *implicitly* with *p*_*u*_ in the texts by recursively triggering the appropriate premises constructed in step 1 and the given premises extracted in step 2 using the standard rules of inference for predicate logic. The conclusion will be a functional category that co-occurs implicitly with *p*_*u*_ in the texts. “Inferring the functional terms that cooccur implicitly with an unannotated protein in texts using predicate logic” section describes this process in detail.

## Methods

### Constructing protein specification rules

#### Representing protein specification rules in a pattern similar to predicate Logic’s premises

A predicate is a statement of one or more predicate variables. It can be transformed to a proposition by assigning values to the variables. These values determine whether the statements are true or false. The propositions are constructed by connecting the statements using logical connectives. PL-PPF composes protein specifications in a similar fashion. Using known protein and biological characteristics, PL-PPF composes the protein specifications from these known characteristics. It represents the specifications in a pattern similar to predicate logic’s premises [14]. It uses these premises to find relations between an unannotated protein and protein functional categories. The specification rules can be updated periodically as new protein characteristics may be discovered. However, the update intervals should not be short, since new protein characteristics are discovered infrequently. We present in Table 1 a *sample* of protein specification rules in the form of predicate logic’s premises. It includes only the rules used in the examples presented in the paper to illustrate the proposed concepts. We constructed the premises in Table 1 based on the following well-known protein characteristics:

- Premise R_1_ is constructed based on the following protein characteristics: (1) the folding of a protein takes place after a sequence of structural changes *(the final stage of folding determines the structure of the protein)* [5], and (2) the structure of a protein defines the function of the protein [5].
- Premises R_2_ and R_3_ are constructed based on the following protein characteristic: each protein’s sequence is unique and defines the structure and function of the protein [1].
- Premise R_4_ is constructed based on the following protein characteristics: (1) the covalent bonds of a protein contribute to its structure [5], and (2) the raw sequence of a protein’s amino acids determines its structure [1].
- Premise R_5_ is constructed based on the following protein characteristic: a protein’s non-covalent interaction folding and dimensional structure can define the protein’s biological function [5].
- Premises R_6_ is constructed based on the following protein characteristic: protein-protein interactions form complexes by interacting with one another [23].
- Premises R_7_ and R_8_ are constructed based on the following protein characteristics: (1) a complex assembly can result in a new function that neither protein can provide alone *(the combined functionalities of the interacting proteins determine the new function)* [23], and (2) the interacting proteins carry out their functions in the complex *(the functions of the individual interacting proteins can be determined from the new complex assembly function)* [23].
- Premise R_9_ is constructed based on the following protein characteristics: (1) proteins can be classified based on the similarities of their structural domains [1], (2) the structure of a protein reveals an insight into its function [5], and (3) the function of a protein *p* can be inferred from the functions of proteins that fall under the same structural classification as *p* [1].
- Premise R_10_ is constructed based on the following protein characteristics: (1) proteins can be classified based on the similarities of their amino acid sequences [5], and (2) the function of a protein *p* can be inferred from the structures of the proteins that fall under the same amino acid sequence classification as *p* [5].
- Premise R_11_ is constructed based on the following protein characteristic: the sequence of a protein’s amino acids is inferred from the combination of the protein’s covalent interactions with ligands and the protein’s function [1].
- Premise R_12_ is constructed based on the following protein characteristic: non-covalent bonds between proteins during their transient interactions lead to Protein-Protein Interactions [18].
- Premise R_13_ is constructed based on the following protein characteristic: the structure of a protein can reveal an insight into its amino acid sequence [5].

**Table 1:** A sample of known protein characteristics represented in a form similar to predicate logic’s premises and used as specification rules. The abbreviations in Table 3 are used in the formation of these premises. R_i_ denotes premise number *i*. The following Logic Symbols are used: “∧” for Conjunction; “∨” for Logical Disjunction; “→” for implies

**R**_1_: FD(P_x_) →(ST(P_x_) →F(P_x_))	
**R**_2_: AAS(P_x_) → ST(P_x_)	
**R**_3_: AAS(P_x_) → F(P_x_)	
**R**_4_: CBND(P_x_, L_y_) ∨ AAS(P_x_)→ ST(P_x_)	
**R**_5_: (FD(P_x_) ∨ ST(P_x_)) → F(P_x_)	
**R**_6_: PPI(P_x_, P_y_) → PCF(P_x_, P_y_)	
**R**_7_: PCF(P_x_, P_y_)→(F(P_x_) →F(P_y_))	
**R**_8_: PCF(P_x_, P_y_)→F(P_x_) ∨F(P_y_)	
**R**_9_: (ST(P_x_) ∧ ST(P_y_)) → (F(P_x_) →F(P_y_))	
**R**_10_: (AAS(P_x_) ∧ AAS(P_y_)) → (ST(P_x_) →F(P_y_))	
**R**_11_: CBND(P_x_, L_y_) ∧ F(P_x_) → AAS(P_x_)	
**R**_12_: NCBND(P_x_ ∧ P_y_) → PPI(P_x_, P_y_)	
**R**_13_: ST(P_x_) → AAS(P_x_)	

#### Extracting biological molecule terms that co-occur explicitly with an unannotated protein in biomedical texts

PL-PPF extracts the biological molecule terms that co-occur *explicitly* with an unannotated protein *p*_*u*_ in the sentences of biomedical texts. If an extracted term denotes a functional category *f*, PL-PPF will assign *p*_*u*_ the function *f*. PL-PPF will also use the extracted term to serve as a *given premise* and apply it as a trigger identifier for the appropriate protein specification rules to infer the functional category that co-occurs *implicitly* with *p*_*u*_ in texts. The co-occurrence of a biological molecule term and *p*_*u*_ in a sentence does not guarantee that this term and *p*_*u*_ are associated. To be associated, the term and *p*_*u*_ have to be *semantically related* in the sentence. We consider a term as semantically related to an unannotated protein, if their co-occurrence probability of being related is significantly larger than their co-occurrence probability of being unrelated in texts. PL-PPF computes the occurrence probabilities of terms using Z-score [32]. For two terms in texts associated with an unannotated protein to be semantically related, the co-occurrences of the same terms in the training dataset stored in PL-PPF’s database should be considered semantically related.

We use the term “training dataset” to differentiate between the following: (1) the set of biomedical texts stored in PL-PPF’s database, and (2) the set of biomedical texts associated with an unannotated protein, whose functions need to be annotated. To differentiate between the two, we call the texts stored in PL-PPF’s database a “training dataset”. In order for two molecule terms in texts associated with an unannotated protein to be semantically related, they have to be semantically related in the texts stored in the database (i.e., the training dataset).

We present below two of the key computational linguistic techniques adopted by PL-PPF to extract the molecule terms that are semantically related to an unannotated protein based on their explicit co-occurrences in the sentences:

- Based on linguistics, two nouns are considered related within a sentence, if they are connected by a pronoun (e.g., “that”, “who”, “which”) [19]. PL-PPF adopts a semantic rule based on the above observation for extracting semantically related biological molecule terms.
- Based on linguistics, two nouns are considered unrelated within a sentence, if they are connected by a preposition modifier (e.g., “whereas”, “but”, “while”) [13, 24]. PL-PPF adopts a semantic rule based on the above observation.

### Inferring the functional terms that co-occur implicitly with an unannotated protein in texts using predicate logic

PL-PPF computes the functions of an unannotated protein *p* implicitly using the following: (1) the protein specification rules *(*i.e.*, premises)* described in “Representing protein specification rules in a pattern similar to predicate logic’s premises” section , (2) the biological molecule terms *(*i.e.*, given premises)* that co-occur explicitly with *p* in biomedical literature and described in “Extracting biological molecule terms that cooccur explicitly with an unannotated protein in biomedical texts” section , and (3) the standard *inference rules* for predicate logic. PL-PPF can infer the functions of *p* by recursively triggering the protein specification rules using the premises (i.e., extracted terms) and the standard *inference rules* for predicate logic. At each recursion, an inference rule is triggered and applied to the premises that have been proven previously. This will lead to a newly proven premise. The final conclusion will be a protein function, which will be considered as the function of *p*. The conclusion is valid, if it has been deducted from all previous premises [30]. Table 2 presents the standard inference rules for predicate logic.

**Table 2:** The standard inference rules for predicate logic

*Rule of inference*	*Name*
¬ *q* *p* → *q* --------- ∴¬*p*	Modus Tollens
*p* *p* → *q* --------- ∴*q*	Modus Ponens
*p* ∧ *q* --------- ∴*p*	Simplification
*p* *q* ------- ∴*p* ∧ *q*	Conjunction
*p* ∨ *q* ¬*p* ------- ∴*q*	Disjunctive Syllogism
*p* ---------- ∴*p* ∨ *q*	Disjunctive Amplification
¬*p* → False ----------- ∴*p*	Contradiction
*p* ∧ *q* *p* → (*q* → *r*) ---------------- ∴*r*	Conditional Proof
*p* → *r* *q* → *r* --------- ∴ (*p* ∨ *q*) → *r*	Proof by Cases
*p* → *q* *q* → *r* --------- ∴ *p* → *r*	Law of Syllogism

We now present case studies in Examples 1 to 4 to show the effectiveness of the deductive inferencing methodology presented in this section. The examples use various biological molecule terms as given premises for inferring the functions of unannotated proteins.

**Table 3:** Notations and abbreviations of the terms used in the formation of the premises presented in Table 1

*Abb.*	*Term*
ST(P_x_)	Structure of protein P_x_
FD(P_x_)	Folding of protein P_x_
L_y_	Ligand y
F(P_x_)	Function of protein P_x_
AAS(P_x_)	Amino Acid Sequence of protein P_x_
CBND(P_x_, L_y_)	Covalent bond between Ligand y and protein P_x_
PPI(P_x_, P_y_)	Protein-Protein Interaction of proteins P_x_ and P_y_
NCBND(P_x_, P_y_)	Non-covalent bond between proteins P_x_ and P_y_
PCF(P_x_, P_y_)	Protein Complex of Functions of proteins P_x_ and P_y_

#### Example 1

Consider that PL-PPF extracted the following terms based on their co-occurrences with an unannotated protein P_u_ in biomedical texts after applying the techniques presented in “Extracting biological molecule terms that cooccur explicitly with an unannotated protein in biomedical texts” section: FD(P_x_) and ST(P_x_) (recall Table 3). Using inference rules, we show how the co-occurrences of FD(P_x_) and ST(P_x_) in texts can be indicative of an implicit mentioning of the function of P_x_ (i.e., F(P_x_)). Therefore, the co-occurrences of FD(P_x_), ST(P_x_), and P_u_ can be indicative of an implicit co-occurrences of F(P_x_) and P_u_. Accordingly, the functions of P_u_ is likely to be similar to F(P_x_). Table 4 shows the inference rules, which conclude that the given premises FD(P_x_) and ST(P_x_) are indicative of F(P_x_).

**Table 4:** Inferring the function of protein P_u_ described in example 1

*Step*	*Reason*
1. FD(P_x_)	Given premise (based on its co-occurrence with P_u_)
2. ST(P_x_)	Given premise (based on its co-occurrence with P_u_)
3. FD(P_x_) ∧ ST(P_x_)	Conjunction using steps 1 and 2
4. FD(P_x_)→(ST(P_x_) →F(P_x_))	Premise R_1_ from Table 1
5. F(P_x_)	Conditional Proof using steps 3 and 4

#### Example 2

Consider that PL-PPF extracted the following terms based on their explicit co-occurrences with an unannotated protein P_u_ in biomedical texts: AAS(P_x_) and AAS(P_y_) (recall Table 3). Using inference rules, we show how the co-occurrences of AAS(P_x_) and AAS(P_y_) in texts can be indicative of implicit mentioning of the functions of P_x_ and P_y_ (i.e., F(P_x_) and F(P_y_)). Therefore, the co-occurrences of AAS(P_x_), AAS(P_y_), and P_u_ can be indicatives of implicit co-occurrences of F(P_x_), F(P_y_), and P_u_. Accordingly, the functions of P_u_ is likely to be similar to F(P_x_) and F(P_y_). Table 5 shows the inference rules, which conclude that the given premises AAS(P_x_) and AAS(P_y_) are indicative of F(P_x_) and F(P_y_).

**Table 5:** Inferring the function of protein P_u_ described in example 2

*Step*	*Reason*
1. AAS(P_x_)	Given premise (based on its co-occurrence with P_u_)
2. AAS(P_y_)	Given premise (based on its co-occurrence with P_u_)
3. AAS(P_x_) ∧ AAS(P_y_)	Conjunction using steps 1 & 2
4. AAS(P_x_) → ST(P_x_)	Premise R_2_ from Table 1
5. ST(P_x_)	Modus Ponens using steps 1 & 4
6. (AAS(P_x_) ∧ AAS(P_y_)) ∧ ST(P_x_)	Conjunction using steps 3 & 5
7. (AAS(P_x_) ∧ AAS(P_y_))→((ST(P_x_)→F(P_y_))	Premise R_10_ from Table 1
8. F(P_y_)	Conditional Proof using steps 6 & 7
9. AAS(P_y_) → ST(P_y_)	Premise R_2_ from Table 1
10. ST(P_y_)	Modus Ponens using steps 2 & 9
11. (AAS(P_x_) ∧ AAS(P_y_)) ∧ ST(P_y_)	Conjunction using steps 3 &10
12. (AAS(P_x_) ∧ AAS(P_y_))→((ST(P_y_)→F(P_x_))	Premise M_10_ from Table 1
13. F(P_x_)	Conditional Proof using steps 11&12

#### Example 3

Consider that PL-PPF extracted the following term based on its explicit co-occurrences with an unannotated protein P_u_ in biomedical texts: ST(P_x_) (recall Table 3). Using inference rules, we show how the co-occurrences of ST(P_x_) in texts can be indicative of implicit mentioning of the function of P_x_ (i.e., F(P_x_)). Therefore, the co-occurrences of ST(P_x_) and P_u_ can be indicatives of implicit co-occurrences of F(P_x_) and P_u_. Accordingly, the functions of P_u_ is likely to be similar to F(P_x_). Table 6 shows the inference rules, which conclude that the given premise ST(P_x_) is indicative of F(P_x_).

**Table 6:** Inferring the function of protein P_u_ described in example 3

*Step*	*Reason*
1. ST(P_x_)	Given premise (based on its co-occurrence with P_u_)
2. ST(P_x_) →AAS(P_x_)	Premise R_13_ from Table 1
3. AAS(P_x_)	Modus Ponens using steps 1 and 2
4. AAS(P_x_) → F(P_x_)	Premise R_3_ from Table 1
5. F(P_x_)	Modus Ponens using steps 3 and 4

#### Example 4

Consider that PL-PPF extracted the following terms based on their explicit co-occurrences with an unannotated protein P_u_ in biomedical texts: NCBND(P_x_, P_y_) and F(P_x_) (recall Table 3). Using inference rules, we show how the co-occurrences of NCBND(P_x_, P_y_) and F(P_x_) in texts can be indicative of implicit mentioning of the function of P_y_ (i.e., F(P_y_)). Therefore, the co-occurrences of NCBND(P_x_, P_y_), F(P_x_), and P_u_ can be indicative of implicit co-occurrences of F(P_y_), and P_u_. Accordingly, the functions of P_u_ is likely to be similar to F(P_y_). Table 7 shows the inference rules, which conclude that the given premises NCBND(P_x_, P_y_) and F(P_x_) are indicative of F(P_y_).

**Table 7:** Inferring the function of protein P_u_ described in example 4

*Step*	*Reason*
1. NCBND(P_x_, P_y_)	Given premise (based on its co-occurrence with P_u_)
2. F(P_x_)	Given premise (based on its co-occurrence with P_u_)
3. NCBND(P_x_, P_y_)→PPI(P_x_, P_y_)	Premise R_12_ from Table 1
4. PPI(P_x_, P_y_) → PCF(P_x_, P_y_)	Premise R_6_ from Table 1
5. NCBND(P_x_, P_y_) → PCF(P_x_, P_y_)	Law of Syllogism using steps 1 and 5
6. PCF(P_x_, P_y_)	Modus Ponens using steps 6 and 7
7. PCF(P_x_, P_y_) ∧ F(P_x_)	Conjunction using steps 2 and 6
8. PCF(P_x_, P_y_)→(F(P_x_)→F(P_y_))	Premise R_7_ from Table 1
9. F(P_y_)	Conditional Proof using steps 7 and 8

## Results and discussion

We implemented PL-PPF in Java and used Prolog as the logic programming language. We ran it on Intel(R) Core(TM) i7 processor and a CPU that has frequency equals 2.70 GHz. The machine has 16 GB of RAM. We ran PL-PPF using Windows 10 Pro. We compared it experimentally with the following five systems: DeepGO [15], IFP_IFC [29], Text-KNN [31], Text-SVM [25], and GOstruct [9, 26]. DeepGO [15] uses deep learning to learn features from protein sequences for the purpose of predicting protein function. IFP_IFC is a system that we proposed previously for predicting the functions of unannotated proteins by employing random walks with restarts on a protein functional network. The nodes of the network denote the functional categories of proteins and the edges denote the interrelationships between them. Text-KNN and Text-SVM use characteristic terms, which are text features obtained from biomedical texts to represent proteins. The two systems assign an unannotated protein *p*_*u*_ the functions of the set *S* of already annotated proteins, if *p*_*u*_ and *S* have similar characteristic terms. The classifier employed by Text-KNN is based on k-nearest neighbour and the classifier employed by Text-SVM is based on support vector machine. In the framework of GOstruct, an unannotated protein *p*_*u*_ is annotated with the functions of a Gene Ontology (GO) term, if this term co-occurs in close proximity with *p*_*u*_ in biomedical texts.

The complete list of specification rules used by PL-PPF in the experiments and the abbreviations of the terms included in the list can be accessed through the following two links, respectively:http://ecesrvr.kustar.ac.ae:8080/plppf/rules.pdf

http://ecesrvr.kustar.ac.ae:8080/plppf/abbreviations.pdf

### Compiling datasets for the evaluation

#### Gene ontology dataset

We compared the systems using GO dataset [11], which contains GO terms as well as proteins annotated with their functions. We extracted a fragment from the biological process ontology that has 70 GO terms. We also extracted a fragment from the molecular function ontology that has 30 GO terms. We downloaded the GO dataset from [11]. The number of downloaded proteins (which are annotated with the functions of the selected terms) is shown in Table 8. We downloaded the PubMed texts associated with the selected proteins based on their entries in [6]. The number of downloaded texts was 577,486. PL-PPF will use these 577,486 texts as a training dataset for extracting the semantically related GO terms to the selected proteins. We considered a term *t* to be semantically related to an unannotated protein *p*_*u*_, if the co-occurrence probability of *t* and *p*_*u*_ using Z-score [32] is greater than “-1.96” standard deviation (with 95% confidence level).

**Table 8:** Number of GO terms and proteins downloaded for the experiments

	*Biological Process*	*Molecular Function*
*Number of GO terms*	70	30
*Number of proteins*	584, 973	604,625
*Number of proteins used in the experiments* ^*a*^	62,386	16,576

^a^ We selected for the evaluations only proteins that satisfy the following: (1) associated with at least one PubMed publication based on their entries in UniProtKB [6], and (2) have experimental evidence code: IC, IDA, IPI, IEP, EXP, TAS, IMP, IGI, or IC.

#### Saccharomyces genome database (SGD)

We also compared the systems using the 6086 SGD dataset [27]. The dataset is a complete information about the yeast proteins. The functions of these proteins have been experimentally determined by manual curation and verified using peer-reviewed process. We downloaded 46,227 PubMed texts associated with the SGD dataset based on their entries in [6].

### Assessing the results returned by the systems through 5-fold cross validation

We divided each of the GO and SGD datasets to five sets. The systems were assessed five times. At each time, a different set of each of the GO and SGD datasets was used for testing and the remaining four sets were used to train the systems. We considered the testing proteins as unannotated and assessed the systems for predicting their functions accurately. We evaluated two versions of PL-PPF: one adopts all the techniques described in this paper and the other adopts only the explicit terms co-occurrence extraction techniques (i.e., without the inference rules described in “Inferring the functional terms that cooccur implicitly with an unannotated protein in texts using predicate logic” section). This will enable us to determine the impact of the inference rules in inferring implicit terms co-occurrences. We assessed the prediction accuracy of each system for identifying the functions of each unannotated protein *p* using the following standard quality metrics shown in Eqs. 1, 2 and 3:

1 $$ Recall\kern0.5em =\kern0.5em {C}_p/{N}_p $$

2 $$ Precision\kern0.5em =\kern0.5em {C}_p/{M}_p $$

3 $$ \mathrm{F}\hbox{-} \mathrm{value}\kern0.5em =\kern0.5em \left(2\;{\mathrm{Precision}}^{\ast}\kern0.5em \mathrm{Recall}\right)/\left(\mathrm{Precision}+\kern0.5em \mathrm{Recall}\right) $$

- *C*_*p*_: The number of *correctly* predicted functions for protein *p*.
- *N*_*p*_: The actual number of correct functions of protein *p*.
- *M*_*p*_: The number of functions predicted for protein *p* by one of the systems.

**Fig. 1:**
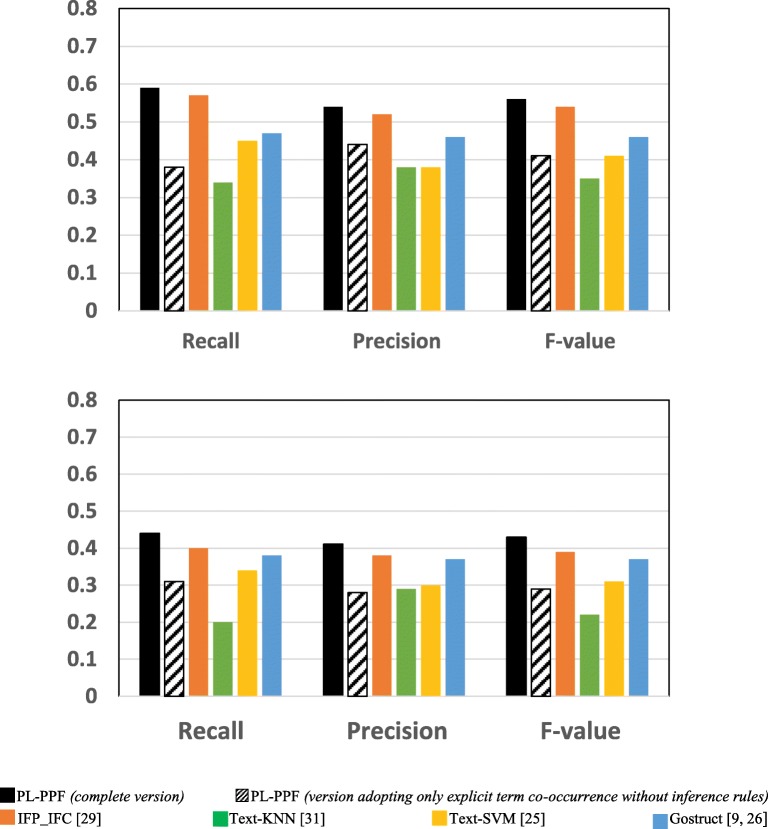
The systems’ performances for predicting GO functions after applying 5-fold cross validation

**Fig. 2:**
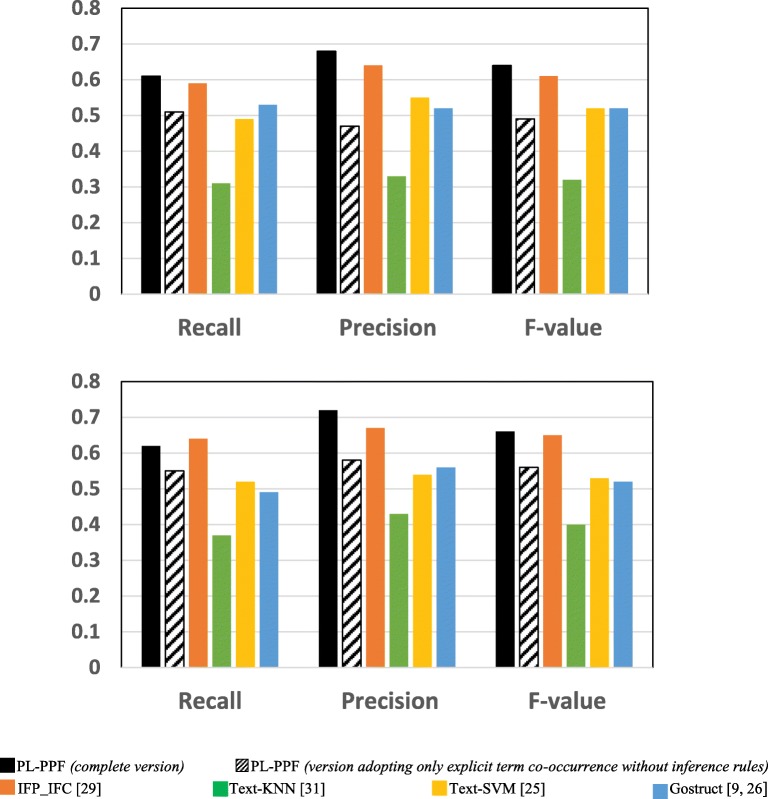
The systems’ performances for predicting SGD functions after applying 5-fold cross validation

Figures 1 and 2 show the results achieved by each system using the GO dataset and SGD datasets respectively. Table 9 shows the number of valid and invalid co-occurrences identified by PL-PPF in the GO and SDG datasets.

**Table 9:** Number and percentage of valid and invalid co-occurrences identified by PL-PPF in the GO and SDG datasets

*Dataset*	*Number and percentage of proteins*	*Biological Process*	*Molecular Function*
*GO* *dataset*	Number of valid co-occurrences identified	39,928	9614
	Number of invalid co-occurrences identified	22,458	6962
	Percentage of valid co-occurrences identified	64%	58%
*SGD* *dataset*	Number of valid co-occurrences identified	2152	858
	Number of invalid co-occurrences identified	1986	1090
	Percentage of valid co-occurrences identified	52%	44%

**Fig. 3:**
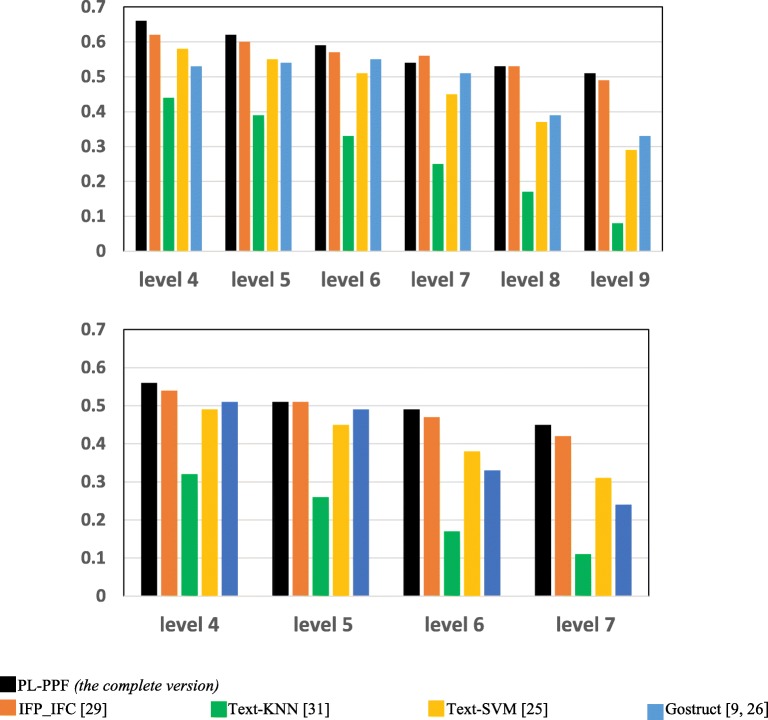
The *Recalls* of the systems for predicting the functional categories of the set of GO terms positioned at the same hierarchical level of the GO ontology

**Fig. 4:**
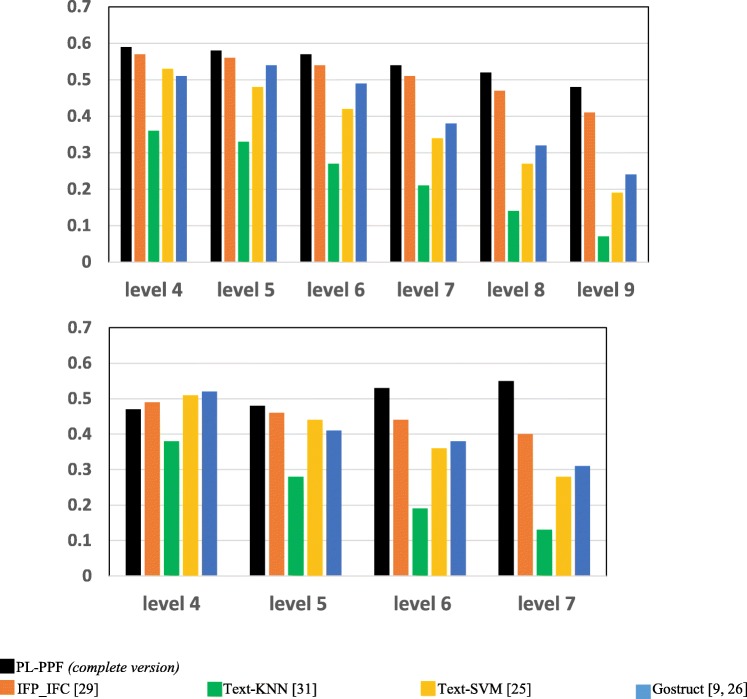
The *Precisions* of the systems for predicting the functional categories of the set of GO terms positioned at the same hierarchical level of the GO ontology

We also assessed each system for accurately inferring the functions of each GO term at different hierarchical levels (depths) of the GO ontology. The size of proteins annotated with the functional category of a GO annotation term decreases as its hierarchical level increases. We aim at investigating whether the accuracy of a system for predicting the functional categories of GO annotation terms gets better as the sizes of these terms increases. We randomly divided the proteins annotated with each functional category *c* into two sets. We considered the proteins in the first set as unannotated, whose functions need to be detected. We considered the biomedical texts associated with the proteins in the second set as a training dataset. We computed the performance of each system for predicting the functions of *c* at different hierarchical levels. Figures 3 and 4 show the results achieved by each system.

### Assessing the results returned by the systems through cumulative-validation

**Fig. 5:**
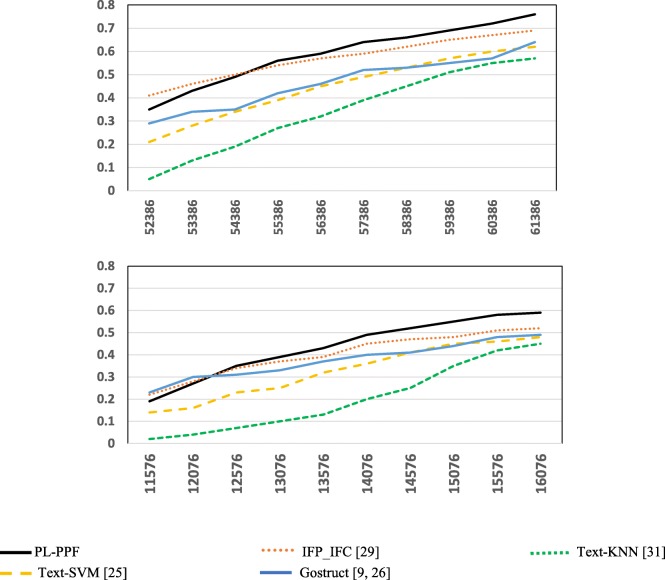
The *Recalls* of the systems for predicting the functional categories of GO terms using a cumulative set of proteins, whose associate biomedical texts are used as a training dataset

**Fig. 6:**
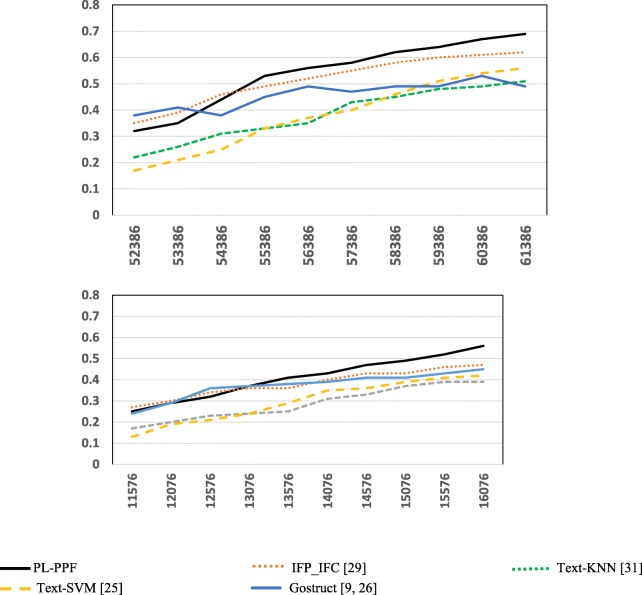
The *Precisions* of the systems for predicting the functional categories of GO terms using a cumulative set of proteins, whose associate biomedical texts are used as a training dataset

We ran each system ten times against the GO dataset. The number of proteins, whose associate biomedical texts are used as a training dataset, keeps accumulating at each run. At each run, we randomly selected 1000 Biological Process testing proteins and 500 Molecular Function testing proteins as unannotated and assessed the systems for predicting their functions. The first run was performed using: (1) 52,386 Biological Process proteins and 11,576 Molecular Function proteins, whose associate biomedical texts are used as a training dataset, and (2) 1000 Biological Process proteins and 500 Molecular Function proteins, whose functions are considered unannotated. At each run, thereafter, the set of proteins, whose associate biomedical texts are used as a training dataset, includes also the Biological Process and Molecular Function proteins, whose functions were annotated in the prior run. Figures 5 and 6 show the results achieved by each system.

### Comparing PL-PPF and DeepGO systems using protein centric maximum F-measure

We compared PL-PPF with DeepGO [15] using protein centric maximum F-measure. DeepGO uses deep learning to learn features from protein sequences for the purpose of predicting protein function. It uses the dependencies between GO Classes to construct the learning model. We followed the same experimental setting used for evaluating the DeepGO method as described in [15]. We also compared the two systems using the same dataset described in [15]. Specifically, we compared the two systems using the following:

1. The protein centric maximum F-measure, which was used in evaluating the DeepGO method.
2. The same GO dataset used in evaluating the DeepGO method (the dataset is shown in Additional file 1: Table S2 of [15]).

**Fig. 7:**
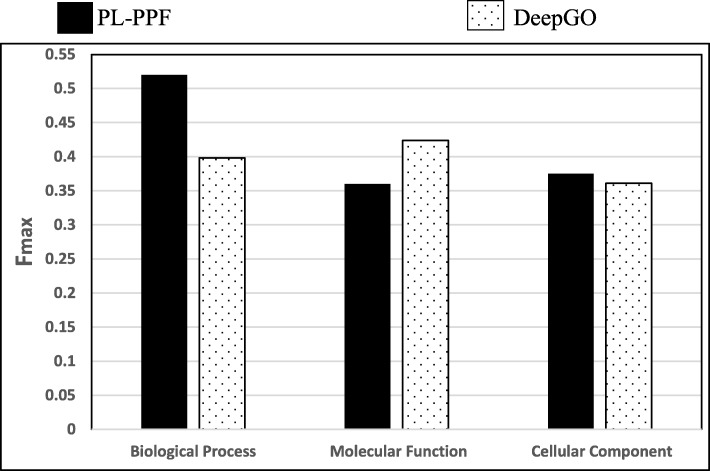
The *protein centric maximum F-measure* of PL-PPF and DeepGO [15] for predicting the functional categories of the GO dataset described in [15]

Figure 7 shows the *protein centric maximum F-measure* of PL-PPF and DeepGO for predicting the functional categories of the GO dataset described in [15].

### Discussion of the results

As Figs. 1, 2, 3, 4, 5, 6 and 7 show, PL-PPF outperformed the other systems. This is an indication of the effectiveness and practical viability of PL-PPF’s combination of explicit and implicit techniques (i.e., its techniques for inferring functional terms that co-occur *implicitly* with proteins using the rules of predicate logic as well as its techniques for extracting functional terms that co-occur *explicitly* with proteins). As the figures show also that the complete version of PL-PPF *(*i.e.*, which employs both of the explicit and implicit techniques)* outperforms significantly the version of PL-PPF, which employs only the explicit techniques. This is attributed to the effectiveness of the rules of predicate logic in inferring the functional terms that co-occur *implicitly* with proteins in biomedical texts.

As Fig. 7 shows, PL-PPF outperformed DeepGO in the GO Biological Process and Cellular Components subontologies. However, DeepGO outperformed PL-PPF in the Molecular Function subontology. Actually, we observed that PL-PPF performs better in the Biological Process dataset then the Molecular Function dataset in all conducted experiments including the ones described in “Assessing the results returned by the systems through 5-fold cross validation” and “Assessing the results returned by the systems through cumulative-validation” sections. We will investigate the root cause of this in a future work.

As Figs. 5 and 6 show, the Recall and Precision values of the systems get better as the sizes of proteins, whose associate biomedical texts are used as a training dataset, increase. However, the Recall and Precision values of PL-PPF and IFP_IFC increase at higher rates. When the set of training texts is small, it would not have enough sentence structures. As a result, PL-PPF cannot accurately determine whether the sentences have solid relationships between their terms. Therefore, as the size of training biomedical texts gets larger, the z-score values computed by PL-PPF for determining semantically related terms become more accurate. This is advantageous for PL-PPF, since the size of biomedical texts associated with proteins in real-world increases significantly over time. As Figs.3 and 4 show, PL-PPF predicts the functions of GO annotation terms at lower hierarchical levels with better accuracy than higher-level ones.

In general, we attribute the performance of PL-PPF over the other five systems to the fact that PL-PPF employs a combination of statistical and logic-based approaches while the other five systems employ only statistical-based approaches. That is, PL-PPF includes a combination of statistical-based *explicit* term extraction component and logic-based *implicit* term extraction component. Our hypothesis is that important biological molecule terms pertaining functional categories are likely to co-occur *implicitly* with proteins in biomedical texts.

Table 10 shows the percentages of valid explicit and implicit terms that PL-PPF identified in the datasets used in the experiments. For each of the GO and SDG datasets used in the experiments, Table 10 presents the percentages of terms in the Biological and Molecular Function ontologies identified by PL-PPF. As the table shows, the percentages of implicit terms that PL-PPF identified are considerable.

**Table 10:** The percentages of valid explicit and implicit terms that PL-PPF identified in the datasets. For each of the GO and SDG datasets used in the experiments, the table presents the percentages of valid terms in the Biological Process and Molecular Function Ontologies identified by PL-PPF

	GO		SDG	
	Biological Process	Molecular Function	Biological Process	Molecular Function
% of explicit terms	72%	64%	62%	76%
% of implicit terms	28%	36%	38%	24%

## Conclusions

Some important biological molecule terms pertaining functional categories may implicitly co-occur with proteins in biomedical texts. Most current information extraction approaches do not take advantage of such implicitly inferred terms and focus solely on explicitly mentioned terms in texts. In this paper, we introduced an information extraction system called PL-PPF. The system predicts protein functions based on both explicitly and implicitly mentioned functional terms in biomedical texts. PL-PPF extracts explicitly mentioned functional terms in texts using computational linguistic techniques that identify semantically related terms in differently structured forms of sentences. It extracts implicitly mentioned functional terms by recursively triggering protein specification rules using the standard inference rules for predicate logic. We compared PL-PPF experimentally with the following five systems: DeepGO [15], IFP_IFC [29], Text-KNN [31], Text-SVM [25], and GOstruct [9, 26]. Results showed that PL-PPF outperformed the other systems in terms of inferring the functions of proteins from both the GO [11] and SGD [27] datasets. We also evaluated the impact of inference rules in inferring implicit functional terms by comparing two versions of PL-PPF: one adopts only the explicit techniques and the other is a complete version (i.e., adopts both of the explicit and implicit techniques). Results revealed that the complete version outperformed significantly the other version. This is attributed to the effectiveness of the rules of predicate logic in inferring implicitly mentioned functional terms in texts.

## Additional file

Additional file 1: The data presented in Tables S1 and S2 is the Biological Process and Molecular Function GO annotation terms used in the experiments as well as the randomly selected sets of training and testing proteins annotated with the functions of these GO terms. (DOCX 56 kb)

## Abbreviations

AAS(P_x_): Amino Acid Sequence of protein P_x_
CBND(P_x_, L_y_): Covalent bond between Ligand *y* and protein P_x_
F(P_x_): Function of protein P_x_
FD(P_x_): Folding of protein P_x_
L_y_: Ligand *y*
NCBND)(P_x_, P_y_): Non-covalent bond between proteins P_x_ and P_y_
PCF(P_x_, P_y_): Protein Complex of Functions of proteins P_x_ and P_y_
PPI(P_x_, P_y_): Protein-Protein Interaction of proteins P_x_ and P_y_
ST(P_x_): Structure of protein P_x_
