# Supplementary material for: Predicting protein functions by applying predicate logic to biomedical literature
Source: BMC Bioinformatics. 2019 Feb 8;20:71. doi: 10.1186/s12859-019-2594-y (PMC6368809; doi:10.1186/s12859-019-2594-y)
Supplement: Supplementary file 1 — The data presented in Tables S1 and S2 is the Biological Process and Molecular Function GO annotation terms used in the experiments as well as the randomly selected sets of training and testing proteins annotated with the functions of these GO terms. (DOCX 56 kb) [file 12859_2019_2594_MOESM1_ESM.docx]

**Additional file 1**

Table S1 The Biological Process GO Annotation Terms used in the Experiments as well as the Randomly Selected Sets of Training and Testing Proteins Annotated with the Terms’ Functions. The Table Shows also the Average Depth (Level) of each GO Term in the Biological Process subontology.

| **GO Term** | **Average depth (level) of GO term** | **Number of Training Proteins** | **Number of Testing Protein** |
| --- | --- | --- | --- |
|  |  |  |  |
| GO:0048856 | 4 | 2130 | 420 |
| GO:0002009 | 4 | 633 | 125 |
| GO:0072088 | 4 | 36 | 9 |
| GO:0035295 | 4 | 1890 | 370 |
| GO:0035239 | 4 | 1304 | 260 |
| GO:0001763 | 4 | 865 | 173 |
| GO:0072001 | 5 | 450 | 90 |
| GO:0009653 | 5 | 1345 | 265 |
| GO:0009888 | 5 | 859 | 171 |
| GO:0048589 | 5 | 1828 | 360 |
| GO:0060562 | 5 | 1212 | 240 |
| GO:0001657 | 5 | 438 | 87 |
| GO:0061138 | 5 | 792 | 158 |
| GO:0060429 | 6 | 528 | 105 |
| GO:0048731 | 6 | 1183 | 225 |
| GO:0072009 | 6 | 86 | 20 |
| GO:0001655 | 6 | 204 | 41 |
| GO:0001822 | 6 | 110 | 30 |
| GO:0072073 | 6 | 84 | 21 |
| GO:0060560 | 6 | 1062 | 200 |
| GO:0072033 | 6 | 61 | 13 |
| GO:0060675 | 6 | 277 | 55 |
| GO:0045165 | 6 | 1379 | 270 |
| GO:0007267 | 6 | 1532 | 290 |
| GO:0030154 | 6 | 1596 | 310 |
| GO:0065008 | 6 | 1400 | 270 |
| GO:0048754 | 6 | 687 | 137 |
| GO:0009887 | 6 | 12 | 4 |
| GO:0044699 | 6 | 1912 | 370 |
| GO:2001141 | 6 | 1731 | 335 |
| GO:0010468 | 6 | 1758 | 340 |
| GO:2000112 | 6 | 1637 | 320 |
| GO:0048513 | 7 | 1107 | 220 |
| GO:0048729 | 7 | 465 | 93 |
| GO:0001656 | 7 | 72 | 18 |
| GO:0060993 | 7 | 109 | 21 |
| GO:0072006 | 7 | 100 | 25 |
| GO:0001658 | 7 | 402 | 80 |
| GO:0061326 | 7 | 309 | 61 |
| GO:0045168 | 7 | 459 | 91 |
| GO:0051094 | 7 | 1768 | 340 |
| GO:0051240 | 7 | 1780 | 340 |
| GO:0022603 | 7 | 1850 | 350 |
| GO:0072087 | 7 | 44 | 11 |
| GO:0090183 | 7 | 345 | 69 |
| G0:0061005 | 7 | 279 | 55 |
| GO:0032835 | 7 | 338 | 67 |
| GO:2000027 | 8 | 631 | 126 |
| GO:0072080 | 8 | 241 | 48 |
| GO:0003338 | 8 | 52 | 13 |
| GO:0044767 | 8 | 1755 | 351 |
| GO:0072028 | 8 | 48 | 12 |
| GO:0006366 | 8 | 1840 | 350 |
| GO:0006355 | 8 | 1804 | 350 |
| GO:0031128 | 8 | 213 | 42 |
| GO:0090184 | 8 | 1717 | 34 |
| GO:0072210 | 8 | 72 | 18 |
| GO:0072215 | 8 | 132 | 26 |
| GO:0077273 | 8 | 199 | 39 |
| GO:0072202 | 8 | 119 | 24 |
| GO:0072207 | 8 | 125 | 25 |
| GO:0072075 | 8 | 183 | 36 |
| GO:0072170 | 8 | 108 | 28 |
| GO:0072234 | 9 | 176 | 45 |
| GO:0072017 | 9 | 104 | 20 |
| GO:0072077 | 9 | 32 | 8 |
| GO:0072078 | 9 | 148 | 38 |
| GO:0072070 | 9 | 147 | 37 |
| GO:0072050 | 9 | 67 | 15 |
| GO:0006357 | 9 | 2025 | 390 |

Table S2 The Molecular Function GO Annotation Terms used in the Experiments as well as the Randomly Selected Sets of Training and Testing Proteins Annotated with the Terms’ Functions. The Table Shows also the Average Depth (Level) of each GO Term in the Molecular Function subontology.

| **GO Term** | **Average depth (level) of GO term** | **Number of Training Proteins** | **Number of Testing Protein** |
| --- | --- | --- | --- |
|  |  |  |  |
| GO:0038023 | 4 | 830 | 210 |
| GO:0009927 | 4 | 51 | 15 |
| GO:0000156 | 4 | 1399 | 350 |
| GO:0005057 | 4 | 1014 | 250 |
| GO:0004888 | 5 | 580 | 140 |
| GO:0015026 | 5 | 109 | 20 |
| GO:0005220 | 5 | 42 | 8 |
| GO:0030594 | 5 | 546 | 130 |
| GO:0000155 | 5 | 1034 | 250 |
| GO:0009881 | 5 | 289 | 70 |
| GO:0008329 | 5 | 136 | 30 |
| GO:0004887 | 5 | 81 | 20 |
| GO:0003707 | 5 | 878 | 220 |
| GO:0004896 | 6 | 130 | 35 |
| GO:0016502 | 6 | 169 | 45 |
| GO:0005035 | 6 | 51 | 10 |
| GO:0016917 | 6 | 198 | 50 |
| GO:0008066 | 6 | 301 | 80 |
| GO:0008158 | 6 | 138 | 35 |
| GO:0008046 | 6 | 58 | 15 |
| GO:0004984 | 6 | 3474 | 870 |
| GO:0035586 | 6 | 207 | 55 |
| GO:0017154 | 6 | 82 | 20 |
| GO:0019199 | 6 | 756 | 190 |
| GO:0042813 | 6 | 141 | 40 |
| GO:0004915 | 7 | 111 | 30 |
| GO:0004908 | 7 | 35 | 10 |
| GO:0004950 | 7 | 210 | 50 |
| GO:0004897 | 7 | 29 | 7 |
| GO:0004904 | 7 | 197 | 45 |
